# Characterizing Dietary Advanced Glycation End-Product (dAGE) Exposure and the Relationship to Colorectal Adenoma Recurrence: A Secondary Analysis

**DOI:** 10.3390/nu15051126

**Published:** 2023-02-23

**Authors:** Maren Sfeir, Elizabeth T. Jacobs, Lindsay N. Kohler, Susan E. Steck, Angela K. Yung, Cynthia A. Thomson

**Affiliations:** 1 School of Nutritional Science and Wellness, University of Arizona, 1177 E 4th St, Tucson, AZ 85719, USA; 2 College of Mel and Enid Zuckerman, School of Public Health, University of Arizona, Tucson, AZ 85724, USA; 3 University of Arizona Cancer Center, University of Arizona, Tucson, AZ 85724, USA; 4 School of Public Health, University of South Carolina Arnold, Columbia, SC 29208, USA

**Keywords:** adenoma, dietary advanced glycation end-products, colorectal cancer, cancer, secondary analysis

## Abstract

Limited studies have evaluated the association between dietary advanced glycation end-product AGE (dAGEs) intake and cancer risk; however, no studies have addressed adenoma risk or recurrence. The objective of this study was to determine an association between dietary AGEs and adenoma recurrence. A secondary analysis was conducted using an existing dataset from a pooled sample of participants in two adenoma prevention trials. Participants completed a baseline Arizona Food Frequency Questionnaire (AFFQ) to estimate AGE exposure. N^Ɛ^- carboxymethyl-lysine (CML)-AGE values were assigned to quantify foods in the AFFQ using a published AGE database, and participants’ exposure was evaluated as a CML-AGE (kU/1000 kcal) intake. Regression models were run to determine the relationship between CML-AGE intake and adenoma recurrence. The sample included 1976 adults with a mean age of 67.2 y ± 7.34. The average CML-AGE intake was 5251.1 ± 1633.1 (kU/1000 kcal), ranging between 4960 and 17032.4 (kU/1000 kcal). A higher intake of CML-AGE had no significant association with the odds of adenoma recurrence [OR(95% CI) = 1.02 (0.71,1.48)] compared to participants with a lower intake. In this sample, CML-AGE intake was not associated with adenoma recurrence. Future research is needed and should be expanded to examine the intake of different types of dAGEs with consideration for the direct measurement of AGE.

## 1. Introduction

Colorectal cancer (CRC) is the third most commonly diagnosed cancer in the US and the second leading cause of cancer mortality [1]. It is estimated that in 2022 in the US, there will be 151,030 new colorectal cancer cases and 52,850 estimated deaths as a result of this disease [2]. It is recommended that men and women over the age of 45 years undergo regular screening for colorectal cancer 1. A proportion of individuals will require colonoscopy-based screening, including higher-risk groups, such as individuals with a prior diagnosis of gastrointestinal polyps [3]. There are different types of polyps that can develop asymptomatically along the inner lining of the colon over long periods of time. Adenomatous polyps (adenomas) are the most common type of polyps and are most prone to becoming cancerous, and their removal is a primary prevention strategy to reduce CRC incidence. About half of the adult screened population will be diagnosed with this specific type of polyp over their lifetime. 

Efforts to reduce the risk of adenomas have been widely explored [4], with a significant interest in modifiable behavioral approaches, including dietary modification [5,6]. Diet is associated with CRC risk [7,8], with evidence of a positive association between the regular consumption of relatively higher amounts of red meat, sugar, saturated fat, alcohol, and adenoma development and CRC risk [9,10]. Higher body adiposity and physical inactivity are also significant drivers of CRC [11,12], while consuming greater amounts of higher-fiber foods, fruits, and vegetables is associated with a decreased risk for CRC [13,14].

There are several postulated mechanisms by which diet may promote adenoma development, including effects on inflammation, DNA damage, oxidative stress, alterations in gut mucosal integrity, changes in the gut microbiota, and exposure to carcinogens [15,16,17,18,19,20]. One dietary exposure that has been sparsely evaluated to date is advanced glycation end-products (AGEs). AGEs are heterogenous compounds that are formed endogenously by nonenzymatic reactions or exist in select foods consumed in the human diet [21]. These compounds are known to promote inflammation and reactive oxygen species that, in turn, promote cancer [22,23]. Within the gut, AGEs can promote inflammation by downregulating antioxidative pathways or promoting dysbiosis related to the gut microbiota [22]. The AGE receptor, RAGE, has been identified in cancer-related pathways suggesting that the intake of these molecules may be associated with adenoma development [24,25]. Foods with high amounts of AGEs include animal-derived products, highly processed foods, and foods that are high in saturated fat [26]. The limited studies to date have suggested an association between dietary AGE (dAGE) intake and cancer risk, specifically breast, pancreatic, and colorectal cancer [27,28,29]. However, there are no data robustly evaluating the role of dAGEs and the risk of adenomas. Identifying a dietary modifiable risk factor that drives precancerous lesions such as adenomas could inform primary prevention strategies for CRC.

The objective of this study was to determine whether dAGEs were associated with the risk of precursor lesions to CRC among adults who previously underwent screening for adenoma and CRC. Specifically, we aimed to develop an AGE database for the Arizona Food Frequency Questionnaire to estimate dAGE intake and exposure among participants in the Wheat Bran Fiber (WBF) and Ursodeoxycholic Acid (UDCA) study cohorts [30,31]. Further, we described food groups associated with greater AGE exposure in this study population to inform future exposure assessments. Lastly, we sought to explore if these relationships varied by age, sex, and body mass index (BMI). We hypothesized that higher dAGE intake among adults participating in these two colorectal polyp prevention trials would be associated with greater odds for adenoma recurrence. 

## 2. Materials and Methods

### 2.1. Study Design & Parent Studies 

A secondary analysis was conducted using data from a pooled sample of participants from two double-blind, randomized controlled trials at The University of Arizona Cancer Center. Participants were recruited between 1990–1995 and 1995–1999 for the WBF and UDCA studies, respectively. All participants were undergoing follow-up screening colonoscopy at gastrointestinal practices located in Phoenix, AZ, and Tucson, AZ. To qualify for this study, participants had at least one adenoma detected (>3 mm) and were removed via colonoscopy within 6 months prior to study enrollment. Inclusion criteria across the trials included adults (male and female) between the ages of 40 and 80 years. The primary outcomes in each study were adjudicated adenoma status at an average of 3.1 years of follow-up. WBF participants were randomized to either a daily wheat bran fiber supplement (13.5 g/day) or a low-fiber supplement (2.0 g/day) [32]. UDCA participants were randomized into either a treatment group (8–10 mg UDCA per kilogram of body weight) or the placebo group [31]. 

### 2.2. Study Sample

**Figure 1:**
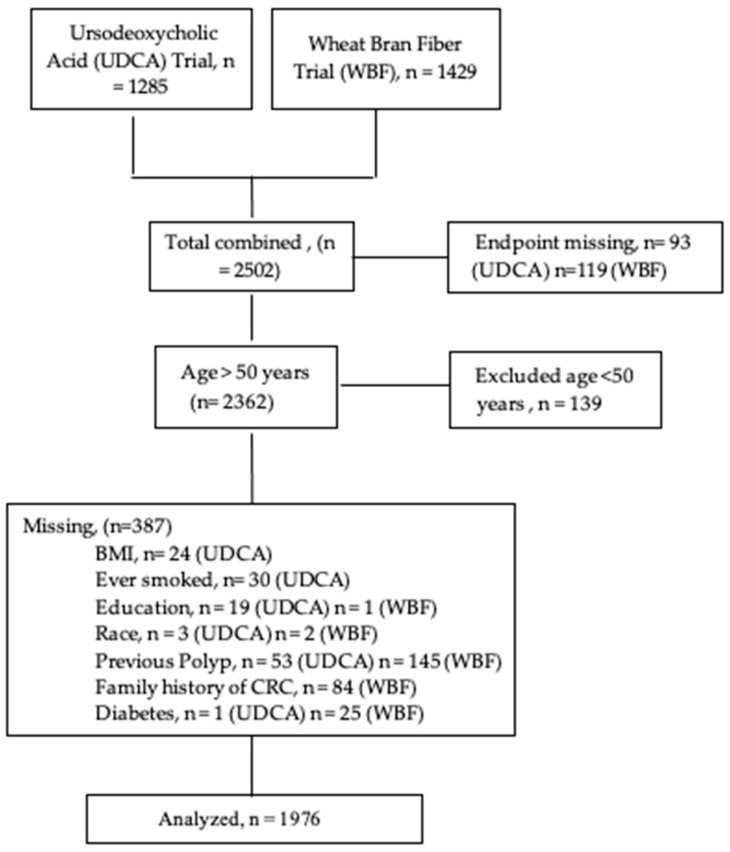
CONSORT Diagram of study participants from the Urosdeoxycholic Acid (UDCA) trial and the Wheat Bran Fiber Trial (WBF). Exclusion criteria from each trial is discussed in the main analysis.

The present analysis was conducted using data collected from 1976 participants, including baseline characteristics, dietary intake, and follow- up for the evaluation of recurrent adenomas or CRC. Additionally, for this pooled analytical sample, we excluded participants under the age of 50 years (*n* = 139), the recommended CRC screening age at the time, to reduce bias being introduced by including individuals undergoing diagnostic screening before age 50 years, as there was likely an underlying condition or symptom that led the participant to seek a colonoscopy. We also excluded participants with any missing data (Figure 1). Both trials were previously approved by The University of Arizona Human Subjects Committee.

### 2.3. Data Collection

Participants in both trials were requested to complete a baseline questionnaire at the enrollment clinic visit. The baseline questionnaire included questions about participant demographics, educational status, marital status, health history, smoking status, NSAIDs use, and history of CRC and/or adenomas. Dietary intake was estimated using the validated Arizona Food Frequency questionnaire (AFFQ). The 175-item food frequency questionnaire assessed food intake over a period of 12 months based on times per day, week, or month [33]. The participant’s physical activity level measured in MET-hours/week (in the past four weeks) was self-reported using the validated 59-item Arizona Activity Frequency questionnaire [34]. 

### 2.4. Data Availability Statement

The data generated in this study are available upon request from the corresponding author. Data will be shared according to the University of Arizona data sharing policy. 

### 2.5. Dietary AGE intake Assessment

**Figure 2:**
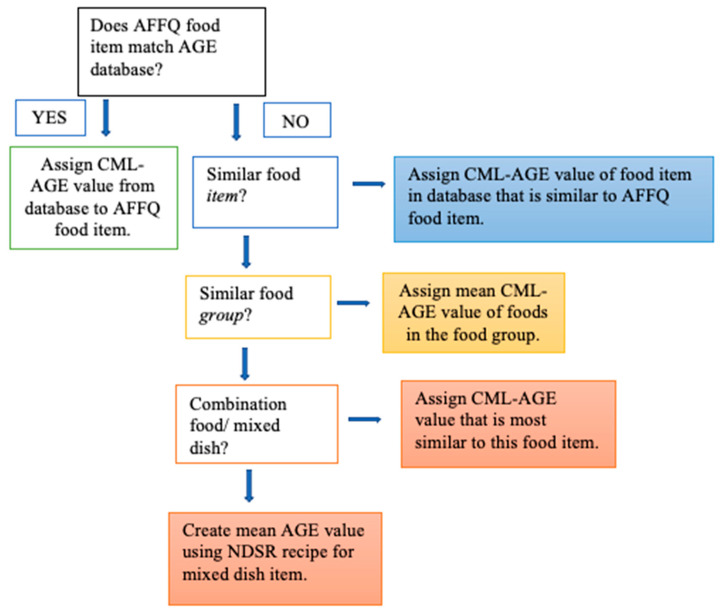
Flow diagram describing the protocol for CML-AGE database assignment to AFFQ food item. Food items within the AFFQ were assigned CML-AGE values using a published database. Food items from the AFFQ that weren’t found in the published data base were assigned average CML values.

Uribarri et al. developed an AGE food composition database item [26] by estimating NƐ-carboxymethyl-lysine (CML)-AGE levels in over 500 food items using an enzyme-linked immunosorbent assay (ELISA) based on a monoclonal anti-CML antibody [35]. CML-AGE is one of many identified AGEs and has been the most commonly measured and studied to date [36]. To estimate dietary AGE intake in our sample, we applied an FFQ food mapping approach similar to that used in other studies [27]. Using the published AGE database [26], CML-AGE values were assigned and quantified to foods in the AFFQ from both trials to estimate dAGE exposure (Figure 2). This mapping process was completed by two independent investigators (M.S. and A.Y.), and discrepancies were resolved with additional input from L.K. AFFQ food items not found in the AGE database were assigned average CML-AGE values created with the existing food items within the same food group. For example, since the AFFQ food item “onions” does not exist in the AGE database, it was assigned an average CML-AGE value of vegetables included in the database. Combination/mixed dishes were assigned CML-AGE average values that were created by assigning CML-AGE values to the individual food components that comprised the recipe of the combination/mixed dish. Recipes for that food item were obtained from the Nutrition Data Systems for Research (NDS-R) software [37] which contains information about nutrient intake, ratios, and serving size. Food items from the AFFQ with different options for cooking methods were assigned an average CML -AGE value across cooking methods. 

### 2.6. Outcome Variable: Adenoma Recurrence

The primary outcome variable for this analysis was colonoscopy-detected adenoma recurrence. To collect recurrent adenoma or adenocarcinoma characteristics, medical records, and pathology reports were reviewed by pathologists in both trials. Adenocarcinoma recurrence was classified as a binary outcome: yes or no as to whether a recurrent adenoma was present during a follow-up colonoscopy. Additional characteristics of recurrent adenomas that were collected for evaluation included the average of the largest adenoma size (mm), adenoma size (>1 cm), number of adenomas, location, histology, and any advanced adenoma recurrence [30,31]. New CRC cases (*n* = 14) after screening adenoma resection were classified as advanced lesions/recurrent adenomas for these analyses and were given a small number. 

### 2.7. Statistical Analysis

Descriptive statistics included the means and standard deviation (SD), which were estimated for continuous variables, while frequencies and percentages were used for categorical variables related to demographics, health history, and lifestyle factors (e.g., diet, physical activity, BMI, tobacco use, and aspirin use). All foods contributing to AGE in the diet were examined to determine the percent of CML-AGE contribution across food categories. For example, fruits, vegetables, and nuts were grouped based on having low CML-AGE amounts. 

AGE exposure was evaluated in relation to the outcome of the presence of at least one adenoma on repeat colonoscopy and controlling for literature-based confounders in relation to factors that drive adenoma risk or AGE exposure. The following covariates were included in the statistical models: age, sex, trial participation (WBF or UDCA), educational status, race, ethnicity, history of polyps previous to qualifying colonoscopy, BMI, family history of CRC, energy intake (kcal/d), tobacco use, and health history, including diabetes.

To determine the association between dAGE intake and adenoma recurrence in participants, a series of logistic regression models were fitted with the binary outcome of adenoma recurrence (yes or no). dAGEs were categorized into quartiles, with the first quartile serving as the referent group. Three models were examined, an adjusted model, a simple model adjusting only for age and sex, and a multivariable model additionally adjusting for BMI, energy intake, study and trial arm, having had a previous polyp prior to study entry, years of formal education, race, smoking history, a family history of CRC, and diabetes. As a sensitivity analysis, physical activity (METs), which was recorded for only 1479 participants, was added to the multivariable model. 

To explore whether the association differed by age, sex, or BMI categories, interaction terms were added to the multivariable model. Interactions were retained if they met a threshold of *p* ≤ 0.20 [38]. In all other analyses, a type I error rate of 0.05 was used, and all tests were two-sided. Data management and analyses were conducted with SAS 9.4 (SAS Institute, Cary, NC, USA) and Stata 17 (Stata Corp, College Station, TX, USA).

## 3. Results

**Figure 3:**
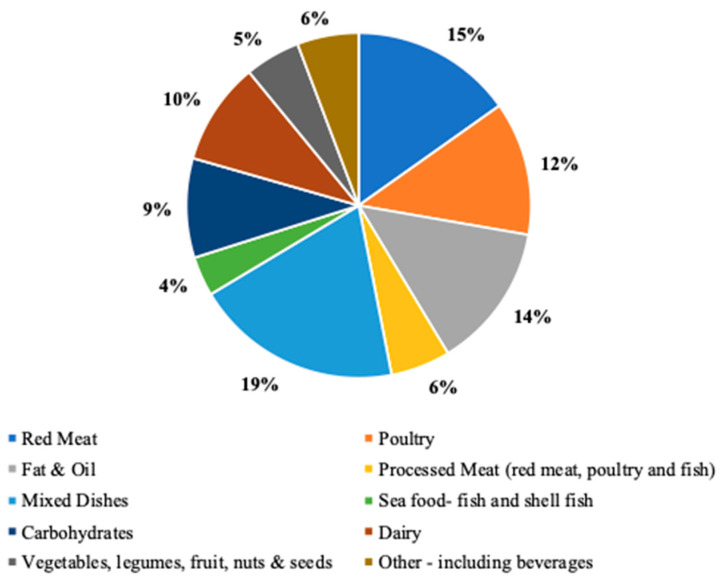
Percent level of food groups that contributed to CML-AGE intake amongst this sample size. Mixed dishes, red meat, fat & oil, poultry, and dairy contributed most in ascending order.

The mean age of the study population was 67.2 ± 7.3 y. Most participants were male (68.1%), white (95.4%), married/cohabitating (83.9%), and had completed at least one year of college (59.3%). Almost half of the population had a history of colorectal polyps prior to qualifying for colonoscopy (45.2%) and had a BMI within the overweight range (25.0–29.9 (kg/m^2^) (44.0%). The mean CML-AGE intake was 5251.1 ± 1633.1 (kU/1000 kcal). The baseline characteristics of 1976 participants were summarized by a quartile of CML-AGE intake (Table 1). CML-AGE intake ranged between 4960 and 17,032.4 (kU/1000 kcal). The majority of male participants were in the highest quartile (88.1%), while the majority of female participants were in the lowest quartile (54.9%) of dAGE exposure. Participants in the lowest quartile of dAGE intake were older (68.9 ± 6.9 y) compared to participants in the highest intake quartile (65.2 ± 7.6 y). Current and prior tobacco use was higher, with a greater reported AGE intake. Participants in the highest quartile of dAGE intake had a higher average daily energy intake (2729 ± 770.4) which correlated with a higher intake of carbohydrates, protein, total fat, polyunsaturated fat, monounsaturated fat, total meat, red meat, processed meat, fruits, vegetables, fiber, alcohol, and added sugar. Higher dAGE intake was observed among participants who were overweight or obese based on BMI. The food groups that contributed most to CML-AGE intake included mixed dishes, red meat, fat/oil, poultry, and dairy (Figure 3). 

A series of unconditional logistic regression models were conducted to determine the association between CML-AGE intake and adenoma recurrence in the sample (Table 2). At trial completion, there were a total of 940 recurrent adenoma cases. There were no significant associations between higher CML-AGE intake and adenoma recurrence in the unadjusted model or simple model adjusting only for age and sex. After adjusting for multiple covariates, there was no significant association between a higher intake of CML-AGE and adenoma recurrence with an OR of 1.02 (95% confidence interval 0.71, 1.48) for participants in the highest quartile compared to the lowest quartile.

We examined the interaction between dAGE exposure and adenoma risk by age, sex, and BMI and observed no significant association. Lastly, we ran a sensitivity analysis adding the physical activity levels (METs) of 1479 participants to the multivariable model and observed no significant association between higher dAGE intake and adenoma recurrence.

## 4. Discussion

We developed a CML-AGE database for the AFFQ to estimate the dietary intake of CML-AGE in a sample of CRC screening-age adults who participated in the WBF and UDCA cohorts. We found no association between self-reported CML-AGE intake and adenoma recurrence after an average of 3.1 years of follow-up. 

Using a published AGE database, we applied a standardized protocol to assign CML-AGE values to food items in the AFFQ and estimated dietary exposure based on the participant’s frequency and quantity of intake at the baseline time point for each randomized controlled trial. In our sample, the consumption of ‘mixed dishes’ contributed most to total dAGE intake, followed by red meat and fat/oil. This may have been influenced by the region in which participants live. Our category for ‘mixed dishes’ predominantly included southwestern food items such as tamales, enchiladas, and burritos. The ingredients that make up these dishes are food items that are high in AGEs, such as meat, animal fat, and dairy. Foods that contributed least to total dAGE intake were fruits, vegetables, and carbohydrates, as has been described in other studies [26]. Overall, our mean reported CML-AGE intake (5251.31 ± 1633.06 (kU/1000 kcal) was less than the average intake reported by Omofuma et al. [27] and similar to the mean intake for women reported by Jiao et al. [29].

These results are the first to evaluate dAGE in relation to adenoma recurrence: a precursor and risk factor for CRC. A few studies have examined the association between dAGE intake and cancer risk overall or CRC. In an analysis of the prospective NIH-AARP Diet and Health study, a positive association between dAGE intake and the risk of pancreatic cancer in men was observed [29]. Omofuma et al. also observed a positive association between dAGE intake and breast cancer risk in prostate, lung, colorectal and ovarian cancer screening trials (PLCO) [27]. Recently, Omofuma et al. examined the association between post-diagnosis CML-AGE intake and mortality among breast cancer patients and observed an association between higher dAGE intake with a higher risk of mortality [39]. Aglago et al. examined the dietary intake of three major AGEs [CML, N€- carboxyethyllysine (CEL), and NGamma-(5-hydro-5-methyl-4-imidazolon-2-yl)-ornithine (MG-H1)] and the CRC risk in the European Prospective Investigation into Cancer and Nutrition (EPIC) study. Unexpectedly, modest inverse associations between CML and MG-HI intake and CRC risk were observed, while a null association with CEL was reported [28]. Additionally, Córdova et al. assessed the dietary intake of CML, CEL, and MGH1 and the overall risk for specific cancer types in the European Prospective Investigation into Cancer and Nutrition (EPIC) study and reported that these three AGE types were not associated with an overall risk for cancer [40]. 

The null findings related to CML-AGE intake and risk for precancerous adenoma formation in the present study could reflect the fact that only certain types of AGEs are involved in adenoma development. The binding of AGEs to RAGEs can induce oxidative stress and inflammation, both of which are characteristics of an adenoma-promoting environment [16,41], but this varies by AGE type. We quantified CML-AGE, a prevalent AGE in vivo [42]. CML-AGEs are considered protein-bound AGEs, which tend to have a higher molecular weight [22,43]. High-molecular weight AGEs need to be broken down within the gut to be absorbed. AGEs with a larger structure, such as CML-AGE, are broken down into two subgroups. The involvement of these subgroups in different mechanisms within the gut is not well understood. However, it may be possible that the interaction between the subunits of CML-AGE and RAGE could be impacted by different factors, such as the gastrointestinal environment. Studies have suggested that AGEs may alter gut microbiota and promote the adenoma development environment [44,45]. Our null findings may also reflect the insufficient sample size to detect the relatively modest effects of AGE on adenoma recurrence. Future studies should evaluate this association using an even larger pooled sample and expand to other dietary AGEs, perhaps including the potential interaction with the gut microbiota. An additional explanation for the null findings may be that other risk factors dominate in promoting recurrent adenomas, thus attenuating the risk associations observed with dAGE intake in this study population. For example, the population that had previously detected adenomas was older, the majority were male, and the number of adenoma recurrences was high (approximately 48%). Lastly, adenomas are initially benign unless they are advanced, in which case, they are more likely to become cancer if not removed in time. It is possible that AGEs may not be involved in the stage of progression from adenoma to cancer but may be involved during initiation, adenoma development, or progression to a more advanced adenoma. 

A strength of this study is the availability and access to complete dietary exposure and adjudicated adenoma outcome data from two cohort trials with a relatively large, combined study sample. These data included robust information on relevant covariates, including baseline demographics, overall dietary intake, physical activity, and health status. Our dataset expanded the earlier food database by providing the estimated AGE levels in combined food items (e.g., burritos, enchiladas, tamales) that were not included in the dataset developed by Uribarri et al. Furthermore, we applied a literature-informed protocol to create an AGE dataset for the AFFQ, which could be used for future analyses. 

However, limitations exist, including the self-reported nature of dAGE exposure. There is a lack of valid biomarkers of dAGE at this time. The enzyme-linked immunosorbent assay (ELISA) is a method to measure serum levels of endogenous AGEs but was not available in our studies [46]. We also recognize that the lifestyle and behavior of participants at the baseline may not be an accurate representation of risk exposures over the longer time course of adenoma and/or CRC development, including during the average 3.1 years of follow-up herein. Further, the mean follow-up of 3.1 years may not be long enough to detect an impact from dAGE. Finally, as mentioned above, risk factors for colorectal adenoma and CRC may differ, and conclusions about the relationship between AGEs and CRC cannot be drawn from this study. 

## 5. Conclusions

Our findings do not support our initial hypothesis that dAGE intake among this pooled sample of adults with previous adenomas is associated with adenoma recurrence. Future studies should consider larger samples with longer-term follow-up and possibly biomarkers of dietary exposure to AGEs. In addition, future studies should consider evaluating younger participants as AGEs may be involved in the initiation of adenoma development. Further, exploring additional dietary AGEs could shed light on whether other AGEs could be involved in precancerous adenoma development.

## Figures and Tables

**Table 1:** Baseline characteristics of pooled sample by quartiles of CML-AGE (kU/1000 kcal) intake.

	Quartile 1 *n* = 494	Quartile 2 *n* = 494	Quartile 3 *n* = 494	Quartile 4 *n* = 494
Average CML-AGE intake (kU/1000 kcal)	<4126	4126–5470	5471–6475	>6475
Age (yr)	68.9 ± 7.0	67.8 ± 7.2	66.8 ± 7.2	65.2 ± 7.6
Male	223 (45)	306 (629)	381 (77)	435 (88)
Female	271 (559)	188 (38)	113 (23)	59 (12)
Marital Status, n (%)				
Single	14 (3)	6 (1)	10 (2)	9 (2)
Married/Cohabitating	375 (76)	413 (84)	424 (86)	446 (90)
Widow/Widower	73 (15)	43 (9)	32 (7)	21 (4)
Divorced/Separated	31 (6)	32 (6)	27 (5)	18 (4)
Race & Ethnicity, n (%)				
White	468 (25)	480 (26)	477 (25)	461 (24)
Black	6 (0.7)	1 (0.2)	3 (0.2)	2 (0.2)
Hispanic	5 (0.6)	5 (0.5)	7 (0.8)	23 (2.5)
American Indian/Alaskan	4 (0.6)	1 (0.1)	0 (0)	3 (0.2)
Asian	7 (0.6)	3 (0.3)	2 (0.3)	2 (0.2)
Other	4 (0.5)	4 (0.3)	5 (0.5)	3 (0.3)
Physical Activity (MET- Hours/week)	18.22 ± 19.1	19.72 ± 42.9	17.84 ± 34.8	17.28 ± 18.26
Educational Status, n (%)				
Elementary/Primary school completed	5 (1)	14 (3)	8 (2)	19 (4)
Some or all high school	214 (43)	202 (41)	175 (35)	168 (34)
At least one year of college	275 (56)	278 (56)	311 (63)	307 (62)
Family history of CRC *^a^*	107 (22)	104 (21)	100 (20)	112 (23)
Previous polyps *^b^*	231 (47)	216 (44)	222 (45)	223 (45)
Lifestyle Characteristics				
Current smoker	58 (12)	51 (10)	63 (13)	71(14)
Ever smoked	308 (62)	328 (66)	344 (70)	358 (72)
Aspirin *^c^*	144 (29)	144 (29)	168 (34)	132 (27)
BMI category, n (%)				
Underweight < 18.5 (kg/m^2^)	5 (1)	2 (1)	4 (1)	2 (1)
Normal 18.5–24.9 (kg/m^2^)	173 (35)	149 (30)	130 (26)	102 (21)
Overweight 25.0–29.9 (kg/m^2^)	213 (43)	224 (45)	230 (47)	203 (41)
Obese > 30.0 (kg/m^2^)	103 (21)	119 (24)	130 (26)	187 (38)
Health History				
Diabetes, *n* (%)	42 (9)	33 (7)	51 (10)	55 (11)
Dietary Intake mean ± SD				
Average CML-AGE intake (kU/100g)	4960 ± 1170	7902 ± 749	10782 ± 948	17032 ± 4898
Energy, kcal/day	1287 ± 411	1700 ± 448	2073 ± 491	2729 ± 770.4
Carbohydrate intake (g/day)	197.2 ± 78.3	243.8 ± 88.4	282.3 ± 93.6	350.2 ± 127
Protein intake (g/day)	47.1 ± 14.8	62.44 ± 15.3	76.54 ± 16.7	103 ± 28.87
Total fat intake (g/day)	35.4 ± 11.6	52.64 ± 12.6	69.54 ± 15.2	101.5 ± 31.32
Saturated (g/day)	11.2 ± 4.26	16.83 ± 4.89	22.71 ± 6.31	34.06 ± 12.45
Poly-unsaturated fat (g/day)	7.82 ± 3.02	11.38 ± 3.27	14.57 ± 3.95	20.57 ± 6.84
Mono-unsaturated (g/day)	13.4 ± 4.5	20.13 ± 4.98	26.65 ± 6.14	38.78 ± 12.27
Fruit intake (cups /day)	2.04 ± 1.41	2.19 ± 1.67	2.184 ± 1.5	2.329 ± 1.594
Vegetable intake (cups/day)	1.45 ± 0.88	1.759 ± 0.85	2.098 ± 0.98	2.68 ± 1.295
Total meat intake (oz./day)	1.82 ± 0.75	2.74 ± 0.91	3.668 ± 1.2	5.296 ± 2.223
Red meat (g/day)	24.3 ± 13.6	41.01 ± 19.2	57.75 ± 26.2	92.57 ± 48.11
Processed meat (g/day)	5.23 ± 6.44	9.61 ± 9.51	15.26 ± 14.9	23.84 ± 25.18
Total fiber intake (g/day)	17.3 ± 8.52	20.46 ± 9.18	23.15 ± 9.1	27.95 ± 11.1
Alcohol (g/day)	5.1 ± 9.41	7.441 ± 14.5	9.751 ± 19	9.742 ± 14.81
Added Sugar (tsp/day)	7.93 ± 5.37	10.94 ± 6.88	13.32 ± 8.44	17.41 ± 11.74

AGE, advanced glycation end-product; BMI, body mass index; CML, NƐ- carboxymethyl-lysine; CRC, colorectal cancer; *^a^* History of colorectal cancer in one parent or sibling; *^b^* History of colorectal polyps previous to qualifying for colonoscopy; *^c^* Regular aspirin use (in the last 4 weeks prior to enrollment).

**Table 2:** Odds ratios (95% confidence intervals) for the association between CML-AGE intake and colorectal adenoma recurrence.

	Quartile 1	Quartile 2		Quartile 3		Quartile 4	
Adenoma recurrence cases, *n*	236	226		232		246	
Average CML-AGE intake (kU/1000 kcal)	<4126	4126–5470		5471–6475		>6475	
		OR (95% CI)	*p*-value	OR (95% CI)	*p*-value	OR (95% CI)	*p*-value
Unadjusted	Ref	0.92 (0.72, 1.18)	0.52	0.97 (0.75, 1.24)	0.35	1.08 (0.84, 1.39)	0.49
Adjusted *^a^*	Ref	0.89 (0.69, 1.14)	0.80	0.90 (0.69, 1.16)	0.41	1.00 (0.76, 1.31)	0.52
Fully Adjusted *^b^*	Ref	0.91 (0.70, 1.19)	0.52	0.91 (0.68, 1.22)	0.98	1.02 (0.71, 1.48)	0.90

AGE, advanced glycation end-product; CML, NƐ- carboxymethyl-lysine; *^a^* Adjusted for age and sex; *^b^* Adjusted for age, sex, BMI, study arm, previous polyps prior to study entry, educational status, race and ethnicity, tobacco use, family history of CRC, and health history.

## Data Availability

The data described in the manuscript, code book, and analytic code will be made available upon request pending [e.g., application and approval, payment, other].
